# Supplementary material for: Metabolic turnover and dynamics of modified ribonucleosides by 13C labeling
Source: J Biol Chem. 2021 Oct 9;297(5):101294. doi: 10.1016/j.jbc.2021.101294 (PMC8567201; doi:10.1016/j.jbc.2021.101294)
Supplement: Supplemental Figure S1 [file mmc2.pdf]

Figure S1

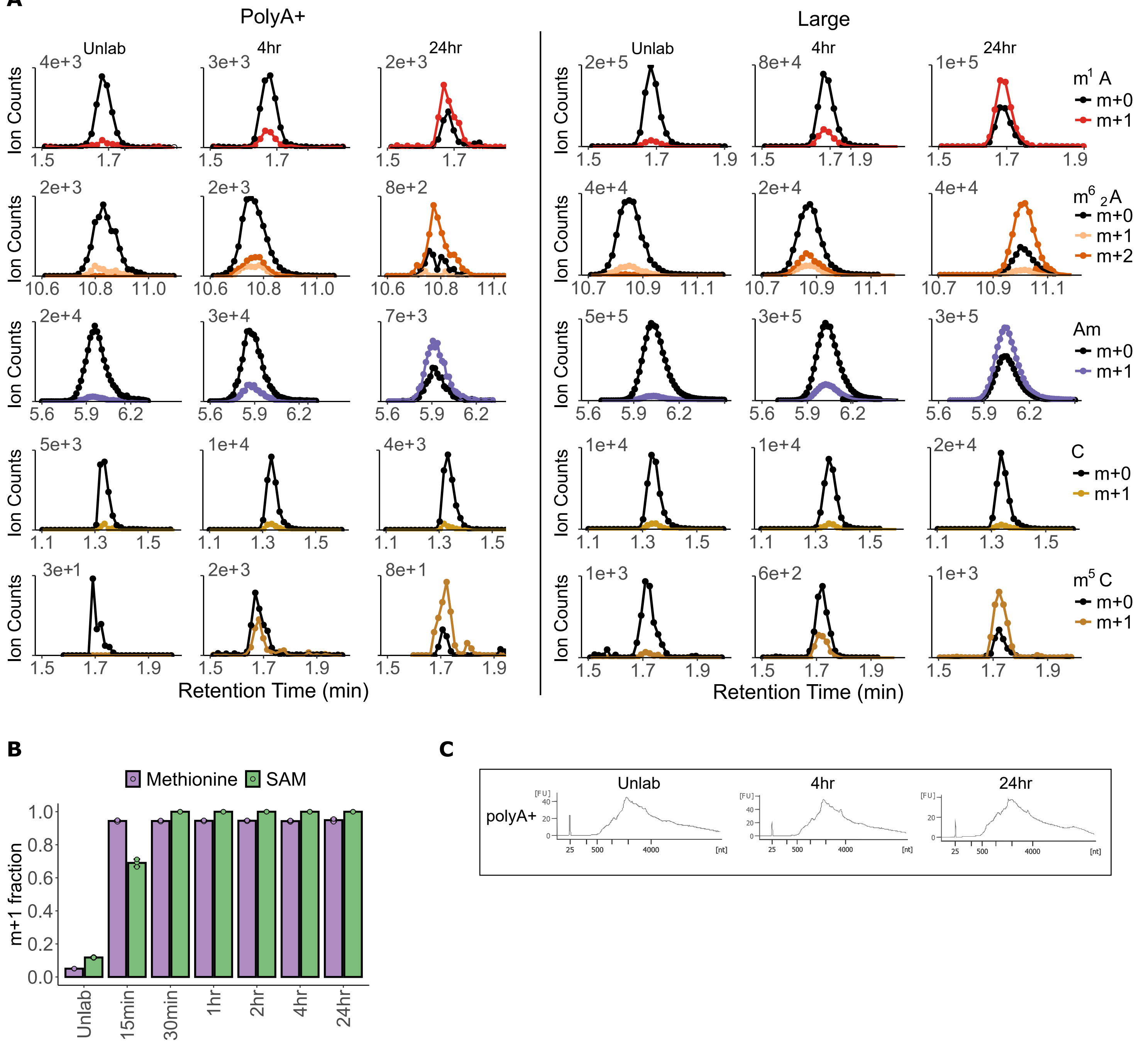

Figure S1. (**A**) The m+0 and m+1 isotopologues of m^{1}A, m$^6_2$A, Am, C and m^{5}C (representative chromatograms) in polyA+ and large RNA following culture with either unlabelled methinine ('Unlab') or [13C-methyl]-methinine for 4 and 24 hours. (**B**) ^{13}C labelling of intracellular methionine and SAM following culture with [^{13}C-methyl]-methinine for the indicated time periods. (**C**) Representative bioanalyzer traces of polyA+ samples. m^{1}A, 1-methyladenosine; m$^6_2$A, *N*6,*N*6-dimethyladenosine; Am, 2′-O-methyladenosine; C, cytidine; m^{5}C, 5-methylcytidine. Error bars represent 90% confidence intervals of three biological replicates in b.
