# Supplementary material for: Metabolic turnover and dynamics of modified ribonucleosides by 13C labeling
Source: J Biol Chem. 2021 Oct 9;297(5):101294. doi: 10.1016/j.jbc.2021.101294 (PMC8567201; doi:10.1016/j.jbc.2021.101294)
Supplement: Supplemental Figure S2 [file mmc3.pdf]

Figure S2

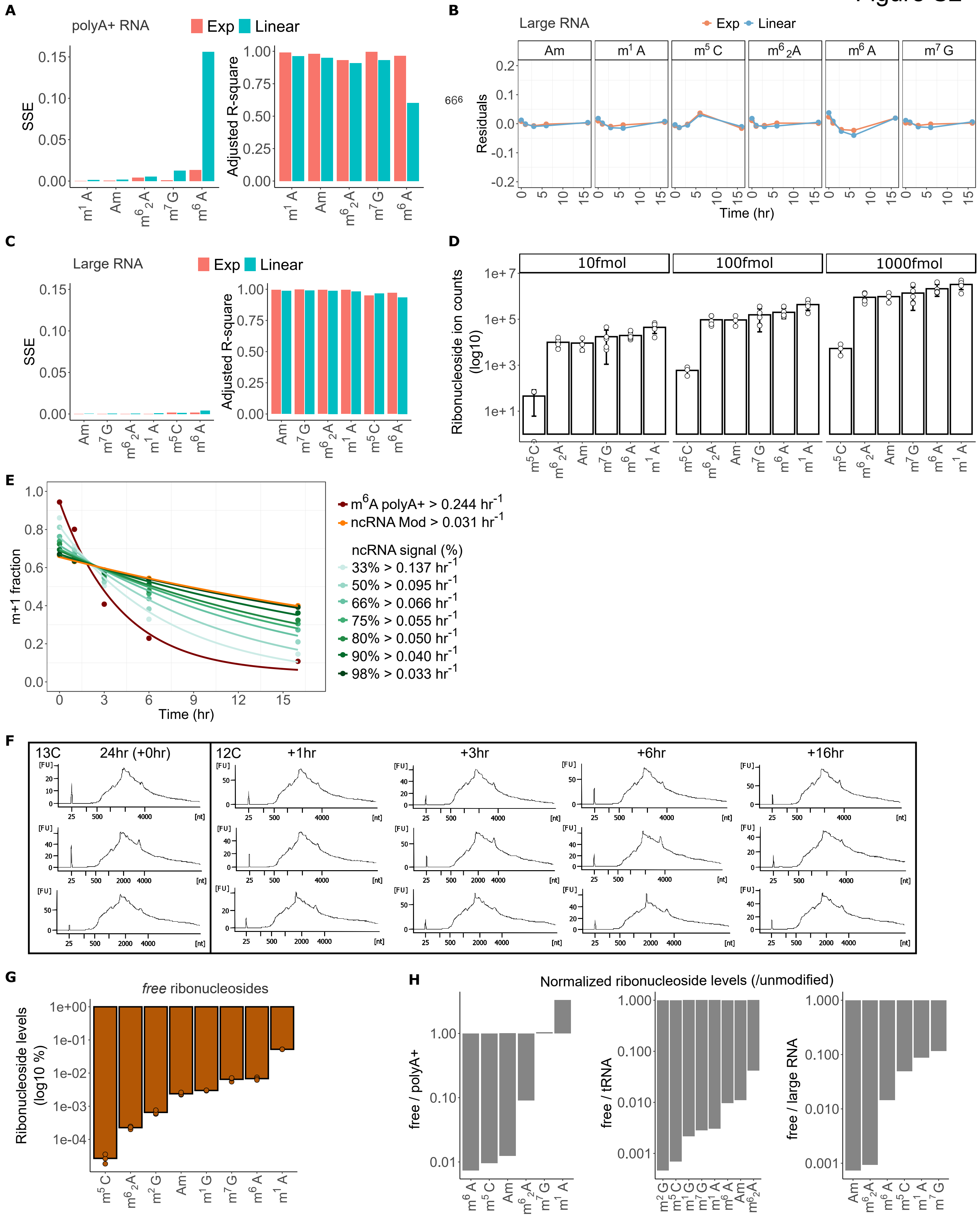

Figure S2. (**A-C**) Goodness-of-fit of a linear vs. exponential fit of isotopologue fractions determined in the chase experiment for polyA+ (A), and large RNA (B-C); SSE, sum of squared errors. (**D**) Ion counts of equimolar analysis of pure ribonucleosides. (**E**) Projected isotopologue fraction of a bona fide mRNA modification as measured in polyA+ RNA, with different degrees of ncRNA contamination. The effect on the isotopologue fraction over time was estimated from combinations of the m^6A turnover in mRNA (0.244hr-1) with that of ncRNA (0.031hr-1, on average). (**F**) Bioanalyzer traces of the polyA+ samples used in the chase experiments. (**G**) Normalized ion counts of modified ribonucleosides relative to the ion counts sum of all ribonucleosides, in the free ribonucleoside pool. (**H**) Ratio of normalised ion counts between free ribonucleosides and the different RNA classes. Error bars represent standard deviation of at least three biological replicates. In all cases, each replicate is the average of two technical replicas.
